# A tumour suppressive relationship between mineralocorticoid and retinoic acid receptors activates a transcriptional program consistent with a reverse Warburg effect in breast cancer

**DOI:** 10.1186/s13058-020-01355-x

**Published:** 2020-11-04

**Authors:** Tram B. Doan, Vanessa Cheung, Colin D. Clyne, Heidi N. Hilton, Natalie Eriksson, Morag J. Young, John W. Funder, George E. O. Muscat, Peter J. Fuller, Christine L. Clarke, J. Dinny Graham

**Affiliations:** 1 Centre for Cancer Research, The Westmead Institute for Medical Research, The University of Sydney, PO Box 412, Westmead, NSW 2145 Australia; 2 Centre for Endocrinology and Metabolism, Hudson Institute of Medical Research, Monash Medical Centre, Clayton, VIC 3168 Australia; 3 Institute for Molecular Bioscience, The University of Queensland, St. Lucia, Queensland 4072 Australia; 4 Westmead Breast Cancer Institute, Westmead Hospital, Westmead, NSW 2145 Australia

**Keywords:** Mineralocorticoid receptor, Retinoic acid receptor, Breast cancer, Gene expression profiling, Warburg effect

## Abstract

**Background:**

The role of nuclear receptors in both the aetiology and treatment of breast cancer is exemplified by the use of the oestrogen receptor (ER) as a prognostic marker and treatment target. Treatments targeting the oestrogen signalling pathway are initially highly effective for most patients. However, for the breast cancers that fail to respond, or become resistant, to current endocrine treatments, the long-term outlook is poor. ER is a member of the nuclear receptor superfamily, comprising 48 members in the human, many of which are expressed in the breast and could be used as alternative targets in cases where current treatments are ineffective.

**Methods:**

We used sparse canonical correlation analysis to interrogate potential novel nuclear receptor expression relationships in normal breast and breast cancer. These were further explored using whole transcriptome profiling in breast cancer cells after combinations of ligand treatments.

**Results:**

Using this approach, we discovered a tumour suppressive relationship between the mineralocorticoid receptor (MR) and retinoic acid receptors (RAR), in particular RARβ. Expression profiling of *MR* expressing breast cancer cells revealed that mineralocorticoid and retinoid co-treatment activated an expression program consistent with a reverse Warburg effect and growth inhibition, which was not observed with either ligand alone. Moreover, high expression of both *MR* and *RARB* was associated with improved breast cancer-specific survival.

**Conclusion:**

Our study reveals a previously unknown relationship between MR and RAR in the breast, which is dependent on menopausal state and altered in malignancy. This finding identifies potential new targets for the treatment of breast cancers that are refractory to existing therapeutic options.

**Supplementary information:**

**Supplementary information** accompanies this paper at 10.1186/s13058-020-01355-x.

## Background

It has long been recognized that breast cancer is a hormone-dependent disease. Treatments targeting the oestrogen signalling pathway are very effective and represent standard treatment for the 70–80% of breast cancers expressing oestrogen receptors (ER). Moreover, ER and the progesterone receptor (PR) are important prognostic factors, generally associated with less aggressive disease features and a more favourable outcome [1]. However, for breast cancers lacking ER and PR, or for those in which resistant disease emerges, treatment options are much more limited, and the outlook is poorer overall. With the exception of HER2-targeted treatments, which exploit the amplification of the human epidermal growth factor 2 gene, in the subset of cases where this is observed, there are limited treatment options available, with cytotoxic chemotherapies the mainstay of disease management. ER and PR are members of the nuclear receptor (NR) superfamily of which there are 48 members in the human [2]. NR play a diverse variety of roles in normal human physiology, spanning fundamental developmental processes, reproductive functions and metabolic homeostasis. They are also implicated in a range of diseases aside from cancer, including obesity, diabetes and cardiovascular disease [3]. Synthetic ligands have been developed targeting a number of NR, and many of these are approved for use in a range of clinical applications. In recent years, there has been considerable interest in the identification of novel targets that are members of the NR family, in the context of breast cancer, that might be targeted for treatment or provide prognostic power in cancers lacking ER or PR.

The mineralocorticoid receptor (MR) is a member of the steroid receptor sub-family of NR and is expressed in many tissues including the kidney, heart, central nervous system and breast [4, 5]. MR can be activated by the primary adrenal cortical steroid hormone, aldosterone (ALDO), as well as the physiological glucocorticoid, cortisol, although MR preferentially binds ALDO in epithelial tissues while in other tissues, including presumptively breast, cortisol is the primary ligand [6]. Progesterone binds MR as an antagonist, with similar affinity as aldosterone, and reaches sufficient levels during pregnancy and in the luteal phase of the normal menstrual cycle to potentially act as an inhibitor [7, 8]. MR is prominently associated with modulation of ion transport and homeostasis, as well as membrane excitability in neurons and muscle cells, tissue responses to injury and the early cell response to stress [9]. However, its breast tissue-specific roles remain underexplored. Structurally, MR is a classical NR, comprising a C-terminal ligand binding domain, central DNA binding domain and long, unstructured N-terminal domain, which is involved in receptor transactivation and stabilization through an N-C interaction [10]. Ligand binding elicits a conformational change, dimerization and recruitment of coregulatory proteins to activation functions in the ligand binding domain and N-terminal region, leading to DNA binding and transcriptional activation. A spectrum of coregulatory molecules interacts with MR and is a likely source of tissue specificity [11]. Only a small subset of the now more than 400 described coregulators have been assayed directly for a mechanistic relationship with MR, suggesting the existence of yet to be described interactions.

There have been few reports of a functional role for MR in the breast [12]. MR has been reported to crosstalk with the progesterone receptor to induce cell adhesion and growth inhibition in breast cancer cells [13]. In our previous study [14] which examined the expression of all 48 members of the NR superfamily in a cohort of normal and cancer breast tissues, we observed that (i) *MR* expression is lower in cancer compared to normal breast tissues and that (ii) *MR* is an independent predictor of metastasis-free survival in tamoxifen-treated breast cancer patients. We have also reported that *MR* is correlated with many NR and coregulators in normal breast tissues, but that there is marked disruption of these associations in breast cancers [15]. Moreover, we have identified *MR* as part of a group of NR with prognostic value in ER-negative (ER−), human epidermal growth factor receptor 2 (HER2)-amplified and basal breast cancer subtypes. These observations suggest a potential role of MR in both the normal breast and breast cancer, which we have investigated in this study through in silico analyses, in vitro experiments and clinical correlations. In particular, in this study, we have studied the correlation between all 48 nuclear receptors and 238 transcriptional coregulators, in normal and malignant breast subsets, to identify relationships between key factors that may indicate novel regulatory roles in the breast.

## Methods

### Human breast tissues

The primary normal breast and breast cancer tissues used for Taqman low-density array expression profiling have been described in detail previously [14, 15]. Data and de-identified breast tissue samples (either fresh frozen tissue or purified total RNA) were obtained from the tissue banks listed in the Acknowledgements. The study was approved by the human research ethics committees of the participating institutions.

Briefly, the cohort studied included 66 individual cases of primary invasive ductal carcinoma (IDC) with associated histopathological grades and clinical information (age at diagnosis, ER, PR and HER2 status, HRT history) and 50 normal breast samples. Thirty-three breast cancer cases were ER positive and 33 cases were ER negative according to immunohistochemical classification provided by the tissue banks, and this was confirmed by examination of *ER* mRNA expression values (as generated by the TaqMan PCR arrays) in the individual samples. Robust cut-offs were imposed on both protein and mRNA measurements for selection of ER status, as previously described [14].

The 4 breast tissue groups in this study were ER+, *n* = 33 (mean age = 58.8 years, age range = 36 to 90); ER−, *n* = 33 (mean age = 53.2 years, age range = 27 to 85); pre-menopausal normal, *n* = 30 (mean age = 37.6 years, age range = 20 to 46) and post-menopausal normal, *n* = 20 (mean age = 60.4 years, age range = 28 to 78). All analyses in this study used the combined normal sample groups (*n* = 50) as the normal breast cohort (mean age = 49 years, age range = 20 to 78).

### TaqMan low-density array

Total RNA was prepared from frozen tissues by homogenization in Qiazol Lysis Reagent (Qiagen), followed by extraction using RNeasy Lipid Tissue Mini Kit (Qiagen) as per the manufacturer’s instructions. Nuclear receptor and coregulator expression was estimated using Taqman low-density array (TLDA) microfluidic cards containing custom gene expression assays for the 48 members of the nuclear receptor family, 238 coregulators and 16 internal controls, as described previously [14, 15]. Assays were run on an ABI 7900HT real-time PCR instrument (Applied Biosystems).

### Cell culture

MCF-7 Tet-On ER+ breast cancer cells, which stably express the reverse tetracycline-controlled transactivator, were from Clontech Laboratories (Mountain View, CA). Cells were maintained in RPMI-1640 growth medium supplemented with 10% foetal bovine serum and 200 μg/ml G-418. MR-inducible MCF-7 cells were generated by stable introduction of the FLAG-MR pTRE-hyg expression construct, expressing Flag-tagged human MR, under the control of a Tet response element-driven minimal CMV promoter, into MCF-7 Tet-On cells with hygromycin selection. MR expression was absent in these cells until induction with 1 μg/ml doxycycline (Dox).

### Western blot analysis

Western blotting was performed with mouse monoclonal antibody MR1-18 (1:1000) (a gift from Celso Gomez-Sanchez, Department of Internal Medicine, University of Mississippi, Jackson, MS). The membrane was incubated with a horseradish peroxidase-linked anti-mouse 2° antibody (1:5000) (Dako, Carpinteria, CA, USA) for 60 min at room temperature. Antibody binding was visualized by chemiluminescence using the ‘Amersham ™ECL Plus Western Blotting Detection System’ (GE Healthcare, UK).

### Gene expression profiling on microarrays

MR-inducible MCF-7 breast cancer cells were cultured with RPMI + 10% charcoal-stripped heat-inactivated foetal calf serum with or without Dox (1 μg/ml) for 48 h with daily media replacement. Cells were then treated for 6 h with the pure MR agonist, aldosterone (10 nM), all-trans retinoic acid (1 μM), 17β-estradiol (10 nM) or their combination.

Total RNA was isolated using RNAqueous purification columns (Invitrogen). Total RNA (500 ng) was amplified and biotin labelled using Illumina TotalPrep reagents (Invitrogen). The amplified samples (750 ng) were hybridized to human whole genome HT-12v4 gene expression bead arrays using the recommended Illumina reagents and following the manufacturer’s protocol.

### Bioinformatic analysis

#### TLDA gene expression analysis

TLDAs were analysed by the relative quantification method of DCt [14]. The geNorm algorithm [16] in the Integromics StatMiner software package was used to select the most stable housekeeping genes to be used as a reference for normalization. The Ct values of each assayed gene were then normalized against the median of the selected housekeeping genes to obtain the delta Ct (DCt) values for NR coregulators. DCt values previously obtained for the 48 NR in [14] were used to perform combined analyses of the expression correlation pattern of nuclear receptors and coregulators. Ct value measurements above 35 cycles were considered inaccurate. Samples for which more than 40% of genes had Ct values above 35 were deemed to be outliers and removed from subsequent analyses. Three breast cancer samples (two ER negative and one ER positive) were removed from both the nuclear receptor and coregulator TLDA data.

#### TCGA

Whole transcriptome expression profiles of 988 primary breast tumours and 106 adjacent normal breast tissues were obtained from the Breast Invasive Carcinoma dataset of The Cancer Genome Atlas (TCGA). The gene-based ‘scaled estimate’ value provided in TCGA’s RNA-SeqV2 data was taken as the expression measure for each gene. In all analyses, this value was transformed by multiplying by 10^6^ to obtain a measure in terms of Transcripts Per Million (TPM) followed by a log2 transformation.

#### Illumina whole genome gene expression arrays

Raw data was background corrected, variance stabilized and quantile normalized using the lumiExpresso function from Bioconductor’s lumi package [17]. Differential expression analysis was performed using Bioconductor’s limma package [18]. Genes with FDR-adjusted *P* values ≤ 0.05 and at least 1.5-fold up- or downregulated were taken as differentially expressed.

#### Gene ontology functional analysis

Gene ontology (GO) terms significantly enriched in genes co-expressed with *MR* or retinoic acid receptor-β (*RARB*) in normal breast and breast cancer samples were identified using the Database for Annotation, Visualization and Integrated Discovery (DAVID) v6.8 online tool [19, 20], using a significance cut-off of enrichment *P* value ≤ 0.005 and false discovery rate ≤ 10%.

#### Survival analysis

To assess the prognostic association of combined *MR* and *RARB* status with breast cancer outcome, the METABRIC cohort of breast cancer cases was stratified into groups based on *MR* and *RARB* transcript levels. The METABRIC dataset consists of 1992 breast cancer microarrays (of which 1853 were annotated with survival data) from the METABRIC study [21] profiled on the Illumina HT-12 v3 platform. Illumina HT-12 v3 array data (EGAD00010000210 and EGAD00010000211) were downloaded from the European Genome-Phenome Archive, and the normalized expression values as published in the METABRIC publication were used. The association between *MR* and *RARB* expression with outcome was assessed in the selected cases by Kaplan-Meier analysis. A Kaplan-Meier log rank *P* value ≤ 0.05 was considered to indicate a significant difference in outcome association between two groups.

## Results

### Inter-correlation between MR and RAR indicates potential functional crosstalk in the normal breast

Coregulators are integral to the mechanisms by which nuclear receptors exert their physiological functions. Therefore, we reasoned that identification of changes in NR-coregulator interactions in breast cancer compared to the normal breast would yield potentially useful insights into novel roles of NR in breast cancer biology. Using highly sensitive high-throughput quantitative RT-PCR expression profiles of 48 NR and 238 coregulators [14, 15], we investigated potential NR-coregulator associations based on gene expression correlations. To do this, we applied Sparse Canonical Correlation Analysis (SCCA) [22] to detect independent maximally correlated groups of NR and coregulators in different sample subgroups: pre-menopausal normal breast (*n* = 30), post-menopausal normal breast (*n* = 20), pre-menopausal breast cancer (*n* = 20) and post-menopausal breast cancer (*n* = 46). Within each sample subgroup, SCCA identified distinct, potentially functionally related, combinations of inter-correlating NR and coregulators, analogous to tightly interconnected groups of genes in a gene co-expression network.

**Fig. 1:**
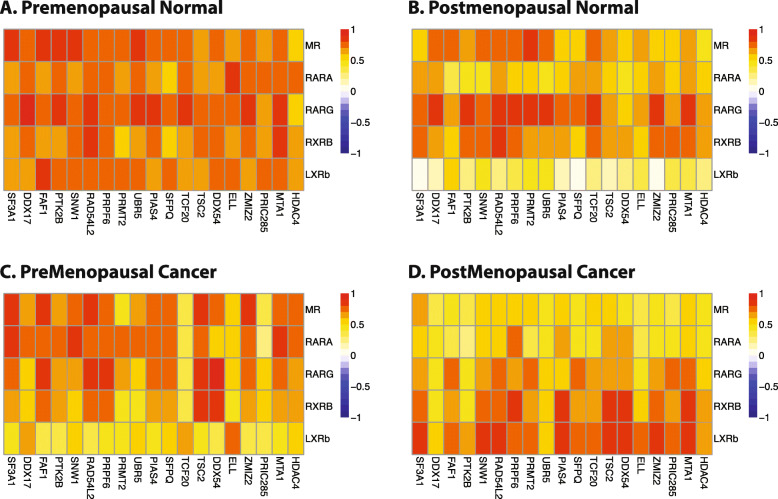
SCCA analysis of NR and coregulator expression in normal and malignant breast. Sparse Canonical Correlation Analysis identified strong inter-correlation between a set of nuclear receptors that include MR and 3 retinoic acid receptors and a set of coregulators in pre-menopausal normal. This strong inter-correlation is decreased in post-menopausal normal, pre-menopausal cancer and post-menopausal cancer. Heatmap colours represent pairwise Spearman rank correlations between each gene pair in the different sample cohorts of the TLDA dataset

SCCA analysis revealed that in pre-menopausal normal breast tissues, the strongest inter-correlation between NR and coregulators was observed between the five nuclear receptors *MR*, *RARA*, *RARG*, *RXRB*, *LXRβ* and a group of 19 coregulators (Fig. 1a). The coregulators involved are known to participate in multiple pathways with various functions including apoptosis, immune response, proteolysis and RNA splicing. The inter-correlation between these nuclear receptors and coregulators was much stronger in pre-menopausal normal samples than in post-menopausal normal breast (Fig. 1b). Strikingly, the correlation between these NR and coregulators was reduced in pre-menopausal cancers (defined as cancers with age ≤ 50 years; Fig. 1c) or post-menopausal cancers (Fig. 1d). This suggested that potential crosstalk between MR and RAR signalling occurring in the pre-menopausal normal breast, at least in the context of coregulator utilization, may be altered in breast cancer.

**Fig. 2:**
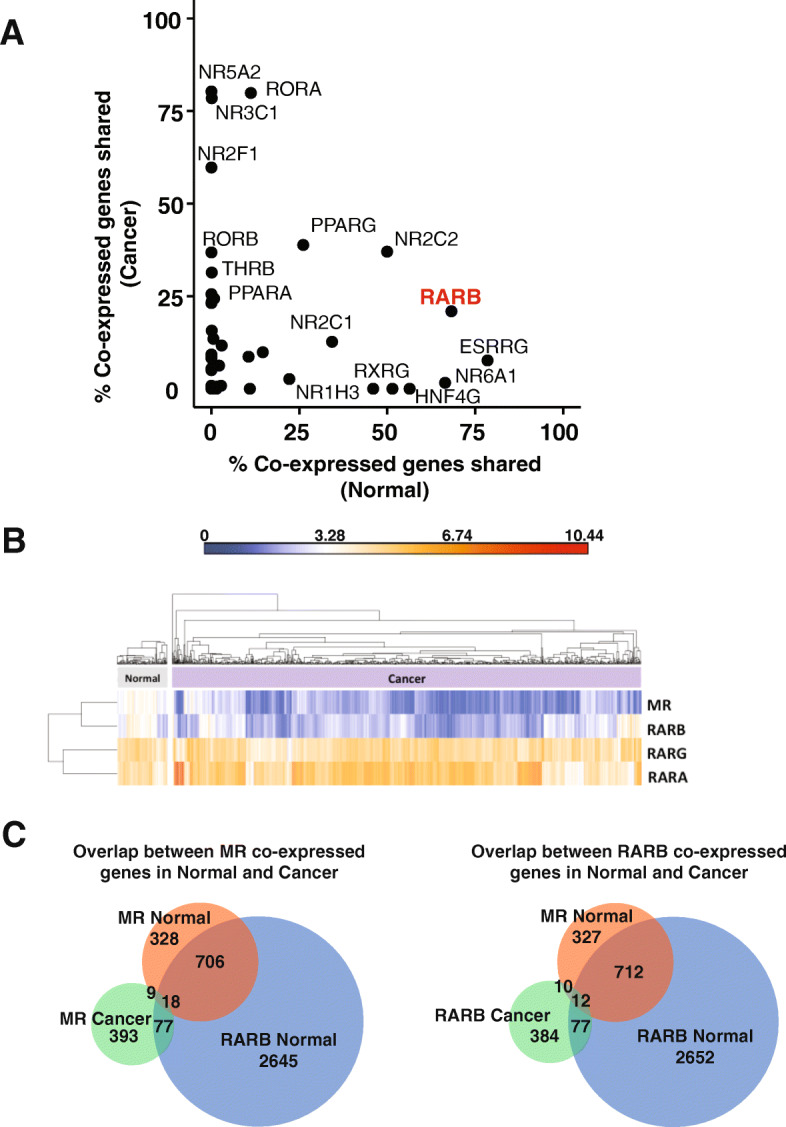
Co-expression-based identification of potential functional crosstalk between MR and RARβ. **a** Percentage of *MR* co-expressed genes shared with other nuclear receptors based on RNA-Seq expression profiles of normal and breast cancer tissues from the TCGA BRCA dataset. **b** Expression of *MR*, *RARB*, *RARG* and *RARA* in normal breast and breast cancer samples of the TCGA RNA-Seq dataset of 106 normal and 988 breast cancer samples. **c** Venn diagrams showing the overlap between co-expressed genes identified in normal and cancer samples for *MR* and *RARB*

As further support for the functional interaction of MR and retinoid receptors in the normal breast, co-expression profiles using RNA-Seq data from TCGA showed that a high percentage of genes co-expressed with *MR* in normal breast tissues were shared with *RARB* co-expressed genes (68%, 724 genes). In cancer tissues, there was a lower percentage of *MR* co-expressed genes shared with *RARB* (104 genes; 21% of *MR* co-expressed genes identified in breast cancer) (Fig. 2a). Hierarchical clustering of the expression of *MR* and the three retinoic acid receptors in the TCGA dataset showed that the expression profile of *MR* more closely resembled that of *RARB* compared to *RARA* or *RARG* (Fig. 2b). The expression of both *MR* and *RARB* was higher in normal breast tissues and decreased in breast cancers while the expression of *RARG* and *RARA* was high and largely invariant in both sample cohorts.

Thus, correlation-based analyses in two different gene expression datasets suggested possible crosstalk between MR and RAR signalling. *RARB* expression was identified as most closely related to *MR* expression in the TCGA dataset while *RARA* and *RARG* were identified as co-correlated with *MR* in the TLDA dataset. These isoform differences may relate to the specific clinical characteristics of the sample cohorts. The pre-menopausal normal cohort of the TLDA dataset consisted of 30 normal breast samples from pre-menopausal women. In contrast, the normal samples derived from the TCGA are histologically normal tissues adjacent to paired tumour samples from all menopausal stages, with the majority being post-menopausal. Given the data shown in Fig. 1, it is likely that the nature of MR crosstalk with different members of the retinoic acid receptors is dependent on menopausal status. Although the TLDA data provided robust measurements of NR and coregulators in a carefully curated cohort, the dataset lacked information on other potentially correlated genes and was limited by the small number of cases. The TCGA data represent transcriptome-wide expression profiles derived from 106 normal tissue samples, for which there is also matched tumour expression profile information, allowing more extensive mining and comparisons to be made. Therefore, the normal samples from the TCGA dataset were chosen for subsequent analyses due to the genome-wide nature of the data and larger cohort size.

### Functional roles of MR and RARB co-expressed genes

To further explore the potential functional roles of MR and RARβ in the breast, genes with expression that was positively or negatively correlated with expression of *MR* or *RARB* were categorized according to whether their expression was correlated with *MR* or *RARB*, and whether the correlation was positive or negative (listed in Supplementary Table 1). The overlap between genes that were correlated with *MR* and those that were correlated with *RARB* was markedly greater in the normal breast than the overlap in breast cancer, and this was reflected in a more substantial overlap in the functional associations with the two NR in the normal breast.

In the normal breast (Supplementary Fig. 1), gene ontology enrichment analysis revealed that *MR* and *RARB* positively correlated genes were enriched with genes involved in cilium assembly, mRNA processing, protein modification (ubiquitination, sumoylation), chromatin modification, cell cycle and DNA damage response (Supplementary Fig. 1A). Gene sets negatively correlated with *MR* or *RARB* in the normal breast were most strongly enriched for genes involved in metabolic processes. In particular, oxidative phosphorylation through the mitochondrial electron transport chain appeared to be enriched in both *MR* and *RARB* negatively correlated gene sets (Supplementary Fig. 1B). Additionally, there were a number of functions associated with genes correlated uniquely with either *MR* or *RARB*, but not both, in the normal breast, and these are also indicated in Supplementary Fig. 1.

In breast cancer (Supplementary Fig. 2), whereas in the normal breast the pattern of co-expressed genes suggested that *MR* and *RARB* shared association with a number of biological processes, many of these connections were lost. We observed a divergence in the biological processes that were overrepresented in the sets of processes associated with *MR* or *RARB* co-expressed genes, although both receptors were co-expressed with genes involved in biological processes important to breast cancer biology. MR positively correlated gene sets were specifically enriched for genes involved in angiogenesis, cell adhesion and response to estradiol (Supplementary Fig. 2A). By contrast, *RARB* positively correlated gene sets were specifically enriched for genes involved in the inflammatory response, apoptosis and protein phosphorylation (Supplementary Fig. 2A). Gene sets negatively correlated with *MR* in breast cancer showed strong enrichment for genes involved in cell cycle regulation while *RARB* correlated gene sets were strongly enriched with genes involved in cellular metabolism (Supplementary Fig. 2B). Both *MR* and *RARB* were positively correlated with genes involved in transcriptional regulation, cell proliferation and signal transduction (GO terms marked with red boxes in Supplementary Fig. 2A).

Confirming the divergence of functions associated with *MR-RARB* in the normal breast and breast cancer, there was a poor overlap of correlated genes of *MR* in normal and cancer (Fig. 2c). The same is observed for *RARB*. By contrast, Fig. 2c also highlights the strong overlap of *MR* and *RARB* correlated genes in normal tissues reflecting the strong overlap in GO terms enriched in *MR* and *RARB* co-expressed genes (as shown in Supplementary Fig. 1).

### Convergence of MR and RAR signalling in the breast

To directly test the convergence of MR and RAR signalling observed in in silico analyses, we constructed an ER+ breast cancer cell line in which MR could be induced. Consistent with the overall lower expression of *MR* in malignant breast tissues, breast cancer cell lines either do not express *MR* or express it at a very low level [23]. We therefore chose the MCF-7 cell line as a well-established, well-characterized model of ER+PR+ breast cancer to construct MR-inducible breast cancer cells. MR-inducible MCF-7 breast cancer cells were treated with either the MR ligand aldosterone (ALDO), retinoic acid (RA) or both, and expression profiling was performed using Illumina human whole genome expression microarrays. Aldosterone rather than cortisol was used as the MR-activating ligand to avoid any confounding effects that might arise from cortisol activation of the endogenous glucocorticoid receptor in MCF-7 cells. Gene expression that changed at least 1.5-fold between two conditions, with a false discovery rate *P* value less than 0.05, was considered to be significantly altered.

**Fig. 3:**
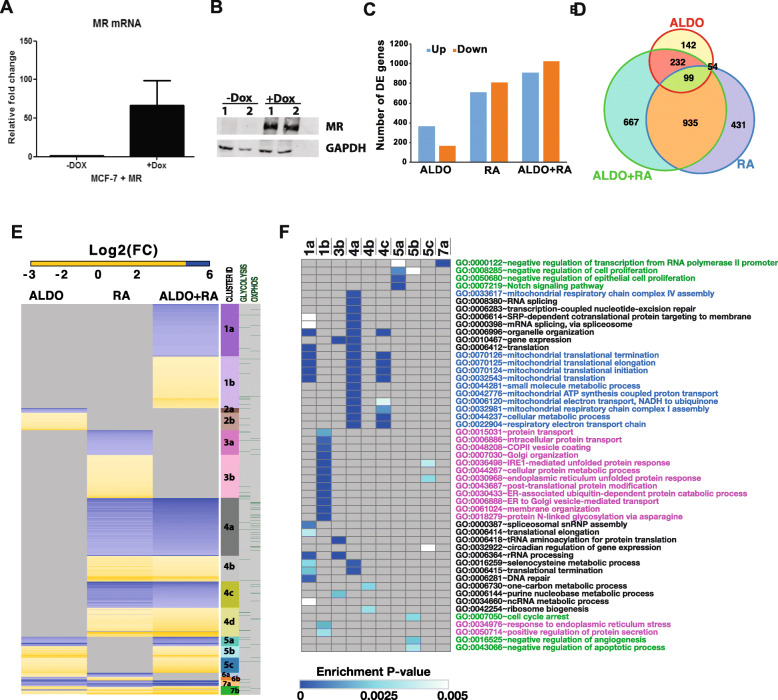
Differentially expressed genes in ALDO-, RA- and ALDO+RA-treated MCF-7 cells. Gene expression profiling of MR-inducible MCF-7 breast cancer cells treated for 6 h with aldosterone (10 nM), all-trans retinoic acid (1 μM) and 17ß-estradiol (10 nM) or their combination and profiled on Illumina’s HT-12 gene expression bead arrays. **a** *MR* mRNA expression, measured by RT-qPCR, uninduced and induced with doxycycline. **b** MR protein expression uninduced and induced with doxycycline. **c** Numbers of genes up- or downregulated in each treatment condition. **d** Venn diagram of the gene overlaps between treatment conditions. **e** Heatmap of the log2 fold change of genes differentially expressed in each treatment condition. Genes are grouped into different gene clusters and annotated with whether the genes are annotated to be involved in the glycolysis or oxidative phosphorylation hallmarks according to MSigDB. **f** Heatmap presentation of Biological Processes GO terms significantly enriched (enrichment *P* value ≤ 0.005 and FDR < 10%) in gene clusters annotated in **e**, only gene clusters with significantly overrepresented GO terms are included in this heatmap. Cell colours represent the enrichment *P* values. Grey indicates no significant GO term was identified

*MR* is robustly expressed both at the mRNA and protein expression level when induced with doxycycline (Fig. 3a and b, respectively). Figure 3c shows the number of genes differentially expressed on ALDO, RA or ALDO+RA treatment in MR-inducible breast cancer cells. Figure 3d shows the overlap of the differentially expressed genes for the three treatment conditions. Figure 3e shows a clustered heatmap of the expression of all genes differentially expressed in any of the three treatment conditions. Differentially expressed genes are grouped into seven clusters and further divided into sub-clusters depending on whether they are up- or downregulated. The genes in each cluster are also annotated according to their association with the Glycolysis or Oxidative Phosphorylation Hallmarks according to MSigDB. ALDO- and RA-regulated genes were largely non-overlapping, except for the small clusters 6 and 7 (Fig. 3e). ALDO+RA treatment resulted in the acquired differential expression of a substantial number of genes that were not regulated when cells were treated with ALDO alone or RA alone (Fig. 3e, cluster 1). This suggests that the expression of these genes is regulated only with convergence of mineralocorticoid and retinoid signalling. ALDO+RA treatment also resulted in loss of differential expression of genes regulated by either ALDO or RA alone (Fig. 3e, clusters 2 and 3).

To better understand the biological processes influenced by ALDO and RA signalling, gene ontology enrichment analysis was performed on each of the gene clusters identified in Fig. 3e and summarized in Fig. 3f. Genes associated with RNA transcription and post-transcriptional processing (GO terms in black) were largely downregulated by RA and ALDO+RA (Fig. 3e, f; clusters 1a and 4a), whereas ALDO and ALDO+RA appeared to regulate gene expression associated with growth inhibition (green GO terms, Fig. 3e, f; clusters 5a and 5b). Co-treatment of ALDO+RA upregulated a set of genes involved in the response to endoplasmic reticulum stress (GO terms in pink, Fig. 3e, f; clusters 1b and 5c), whereas these genes did not show significant expression change with ALDO or RA single treatments. Genes involved in oxidative phosphorylation (GO terms in blue text) were downregulated in both RA single treatment and ALDO+RA co-treatment (Fig. 3e, f, clusters 4a and 4c). Furthermore, many genes involved in glycolysis were upregulated in ALDO, RA and ALDO+RA treatments (Fig. 3e, side panel). Together, ALDO and RA signalling appears to promote glycolysis while suppressing oxidative phosphorylation. This observation suggests that MR and RA signalling together promotes the Warburg effect, which describes a shift towards aerobic glycolysis in preference to oxidative phosphorylation as the major means of ATP generation in cancer cells [24]. However, this could be seen to contradict other evidence suggesting that RARβ is a tumour suppressor gene that inhibits breast cancer migration and growth [25–29]. Since metabolic reprogramming is now recognized as a cancer hallmark [30], we investigated further the effect of MR and RAR signalling on glycolysis and oxidative phosphorylation in breast cancer cells by looking at the expression of key genes involved in the regulation of glycolysis and oxidative phosphorylation.

**Fig. 4:**
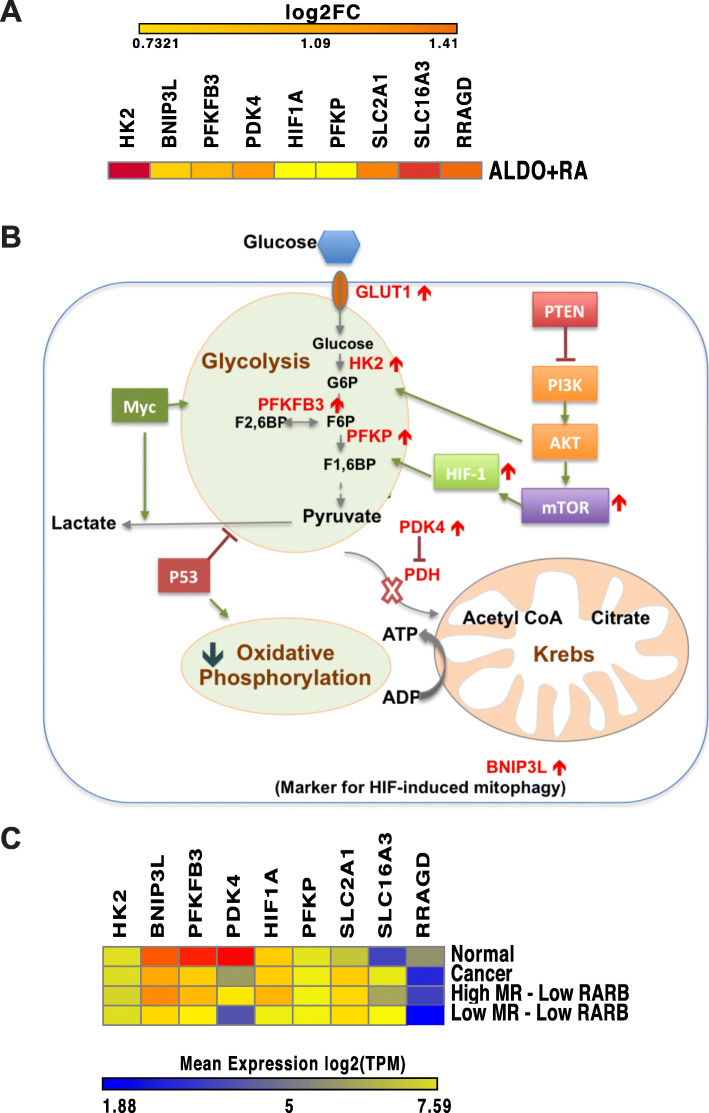
Effect of ALDO+RA treatment on cellular metabolism. **a** The fold change in expression on ALDO+RA treatment in MR-inducible MCF-7 cells for differentially expressed metabolic genes. **b** Schematic diagram of the glycolysis pathways (adapted from [31]), highlighting in red the key genes that show differential expression in the ALDO+RA treatment. **c** Heatmap of the expression level of key genes involved in glucose metabolism in normal samples, cancer samples, cancer samples expressing high level of both *MR* and *RARB* and cancer samples expressing low level of both *MR* and *RARB* from the TCGA breast cancer RNA-Seq dataset

Key pro-glycolysis genes were increased by MR-RAR activation (Fig. 4a). Specifically, on ALDO+RA treatment, we observed an increase in the expression of the *GLUT1* glucose transporter (*SLC2A1*), increased expression of *HIF1A* and genes related to hypoxia response, increased glycolysis and increased expression of *MCT4* (*SLC16A3*) which facilitates lactate export across the cell membrane, as well as an increase in the expression of the HIF-associated mitophagy marker *BNIP3L*. When these effects were overlaid onto a schematic diagram (Fig. 4b) of the key cellular metabolic pathways affecting glycolysis and oxidative phosphorylation in cancer cells, it was noted that in addition to promoting glycolysis and downregulating many genes involved in oxidative phosphorylation, ALDO+RA treatment also upregulated *PDK4*, which is known to inhibit the pyruvate dehydrogenase (PDH) complex, an enzyme that catalyses the oxidative decarboxylation of pyruvate to acetyl-CoA, the input into the Krebs cycle. Inhibiting *PDH* would therefore cause a disconnection between glycolysis and the Krebs cycle preventing ATP generation by the electron transport chain (oxidative phosphorylation). These data are consistent with the view that activation of *PDK4* by MR-RAR would promote glycolysis and suppress oxidative phosphorylation, hence promoting the Warburg effect that has been reported to offer proliferative advantage to cancer cells.

To test the prediction that MR-RAR activation was consistent with increased glycolysis in cancer samples, the genes in Fig. 4a were measured in the TCGA dataset (Fig. 4c). In support of the findings in the cell line, breast cancers expressing high levels of *MR* and *RARB* express higher levels of key pro-glycolysis genes compared with cancers with low *MR/RARB* expression (Fig. 4c).

*PDK4* expression is however higher in the normal breast when compared to breast cancer tissues as shown in Fig. 4c. It can be observed in Fig. 4c that in addition to *PDK4*, the expression profiles of other key pro-glycolysis genes including *BNIP3L* and *BFKFB3* are higher in the normal breast compared to breast cancer tissues. Furthermore, in breast cancer samples expressing high levels of both *MR* and *RARB*, the expression profile of these pro-glycolysis genes is closer to that observed in normal breast than in breast cancer, whereas in breast cancer samples expressing low levels of both *MR* and *RARB*, these pro-glycolysis genes are also expressed at a lower level than observed generally in breast cancer samples. This is a puzzling observation which seems to suggest that in breast cancer, increased glycolysis is not required by breast cancer cells. A caveat may be that expression of some of these genes may reflect adipose-stromal cells which will inevitably be present in normal breast rather than normal or malignant epithelial cell gene expression.

### Effect of MR and RAR signalling on breast cancer cell proliferation and patient survival

**Fig. 5:**
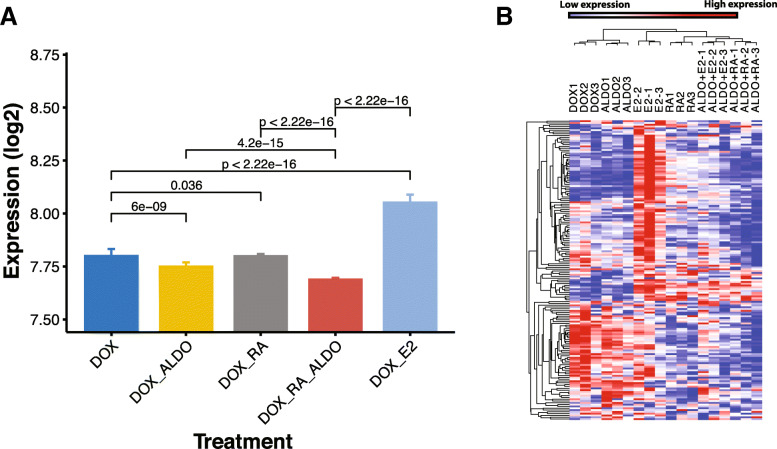
Effect of MR and RAR signalling on breast cancer cell proliferation. The meta-*PCNA* genes which are the top 1% of genes most highly correlated with the proliferating cell nuclear antigen (PCNA), a well-known proliferation marker is used to calculate the meta-*PCNA* index which is defined as the median expression of meta-*PCNA* genes. **a** *PCNA* index as a proliferation measure for MR-inducible cells under different treatment conditions. Here, Dox is used to induce MR; therefore, Dox alone is equivalent to vehicle treatment of MR containing cells. *P* values are based on paired Wilcoxon signed-rank test of the expression of meta-*PCNA* genes for each treatment comparison of interest. **b** Expression of meta-*PCNA* genes under different treatment conditions of MR-inducible MCF-7 breast cancer cells

To test the functional effect of combined MR-RAR activation on breast cancer growth, we investigated the expression of proliferative genes in ALDO-, RA- and ALDO+RA-treated MR-inducible MCF-7 breast cancer cells, using the known proliferative effect of 17β-estradiol (E2) in MCF-7 cells as reference. As an indicator of cell proliferation, we measured the meta-*PCNA* genes (Fig. 5b shows a clustered heatmap of the expression of meta-*PCNA* genes) as well as calculated the meta-*PCNA* index (Fig. 5a). Meta-*PCNA *genes are the top 1% genes most highly correlated with the proliferating cell nuclear antigen (*PCNA*), a well-known proliferation marker in multiple normal tissue types [32]. Figure 5a shows the median expression of the meta-*PCNA* genes for each treatment condition with associated *P* values based on the paired Wilcoxon signed-rank test of the expression of the meta-*PCNA* genes.

Reflecting the known proliferative effect of E2 in breast cancer cells and confirming the activation of proliferative pathways in this cell line model, the *PCNA* index of E2-treated cells was highest (Fig. 5a, where DOX treatment alone, which is used to induce MR, is equivalent to vehicle treatment). This was confirmed by examination of the individual expression of the meta-*PCNA* genes, showing E2 activation of proliferative genes (Fig. 5b, yellow cluster). Neither MR nor RARB activation increased the PCNA index, and in fact, ALDO-treated cells had a lower PCNA index compared to cells treated with DOX alone, and DOX+ALDO+RA-treated cells showed a further decrease in the *PCNA* index (Fig. 5a). E2-mediated expression of the proliferative gene cluster was suppressed on ALDO+E2 treatment, suggesting that ALDO can counteract the E2 proliferative effect when both signalling pathways are active. Furthermore, ALDO+RA co-treatment resulted in further suppression of the meta-*PCNA* genes compared to DOX alone, ALDO alone or RA alone. These decreases were small but highly statistically significant, suggesting that further validation at longer treatment times than 6 h will be important. The second set of proliferative genes (pink cluster) shows a response to E2 and/or RA signalling but not ALDO signalling. Finally, the third meta-*PCNA* gene cluster (blue) contains genes that show a strongly decreased expression on RA/ALDO+RA treatments, but a lesser response to ALDO, E2 or ALDO+E2 treatment.

This result suggests that ALDO and RA signalling can inhibit E2-dependent as well as E2-independent proliferation of breast cancer cells. It also shows that co-activation of ALDO and RA signalling results in a stronger anti-proliferative effect compared to activation of each alone, suggesting a co-operative crosstalk of the MR and RAR signalling pathways to inhibit breast cancer growth.

**Fig. 6:**
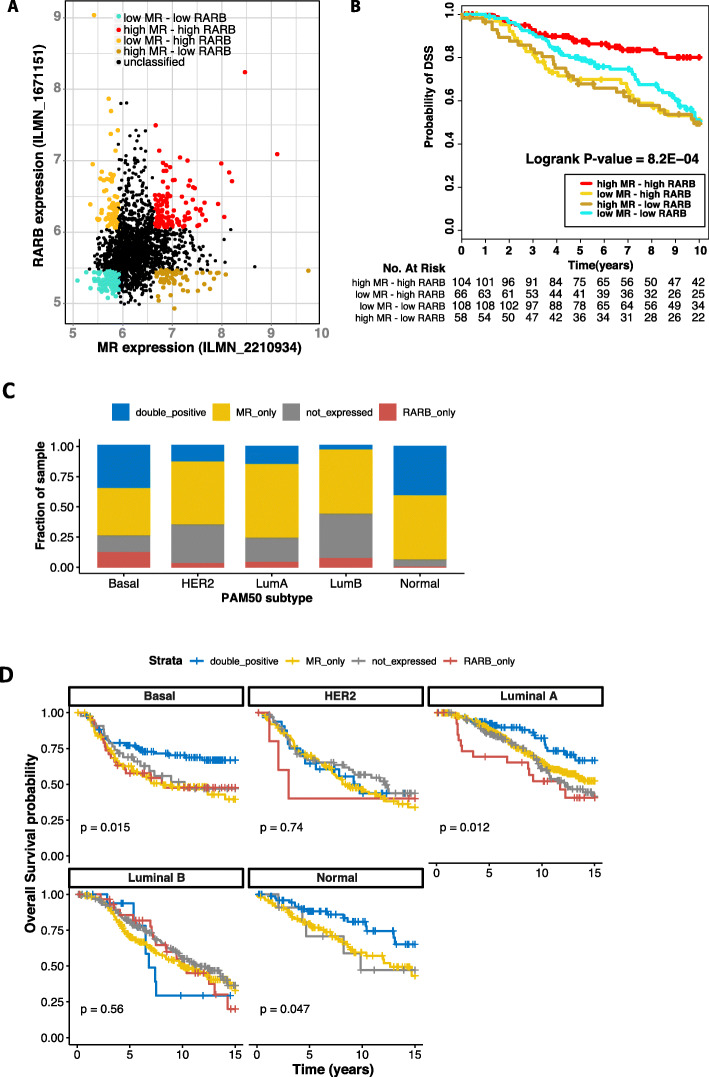
Survival analysis of breast cancer tissues expressing high *MR* and *RARB*. **a** Scatter plot of expression of *MR* against *RARB* in breast cancer samples from the METABRIC dataset. A receptor is classified as highly or poorly expressed in a sample if its expression in that sample is in the top or bottom 20% of its expression range respectively. **b** Kaplan-Meier analysis showing samples expressing a high level of both *MR* and *RARB* have better survival outcome. **c** All breast cancer samples from the METABRIC breast cancer microarray dataset were classified into four categories: double-positive cases express both *MR* and *RARB* based on probe expression cut-off defined in Fig. S3 (blue bars); MR_only cases express *MR* but not *RARB* (yellow bars); RARB_only cases express *RARB* but not *MR* (red bars); and not_expressed cases do not express *MR* or *RARB* (grey bars). The stacked column plot shows the percentage of each of the four categories within each PAM50 subtype from the METABRIC dataset. Here the classification of each breast cancer sample into PAM50 subtype is as defined by the METABRIC publication. **d** Kaplan-Meier plot for each of the four categories of *MR* and *RARB* expression, for all breast cancer samples with survival data in the METABRIC dataset, sub-divided by PAM50 subtype. Due to having only one sample categorized as expressing only *RARB*, there is no survival curve for this category in the normal-like subtype

Given the strong suppressive effect of MR and RA signalling on proliferation in our MR-inducible breast cancer cell model, and the powerful prognostic role of tumour proliferation in breast cancer, we asked whether breast cancers expressing high levels of *MR* and *RAR* would be associated with better survival. Using the METABRIC breast cancer microarray dataset, we first examined the expression levels and degree of variance of *MR*, *RARA*, *RARB* and *RARG* in the cohort. Based on the overall distribution of the expression estimates of all transcripts represented by probes on the Illumina human whole genome array used for this dataset (Supplementary Fig. 3A), we defined any transcript with expression level > 6 to be expressed. Based on this threshold, *RARA* was expressed in all breast cancer cases, whereas *RARG* was detectable in less than 2% of cases in the METABRIC cohort. *RARB* was detected above the threshold in 24% of breast cancers and *MR* was present in 71% of cases (Supplementary Fig. 3B and C). Based on this finding, we explored the association between *MR* and *RARB* dual positivity and breast cancer survival. Breast cancer cases from the METABRIC dataset were classified into four sample subgroups based on the expression of *MR* and *RARB* (Fig. 6a). Kaplan-Meier analysis revealed that breast cancers that were characterized by high expression of both *MR* and *RARB* represented a subgroup with significantly better survival (Fig. 6b). High expression of only one of the two receptors or low or absent expression of both receptors was not associated with equivalently good outcome as their combined high expression (Fig. 6b). This is in agreement with the cell line work and suggests that crosstalk between MR and RAR has a tumour suppressive effect in breast cancer.

In light of this finding and considering that the expression of both *MR* and *RARB* are reduced in breast cancer, we investigated the intrinsic subtype-specific expression level of *MR*, *RARA*, *RARB* and *RARG* in breast cancer cases from the METABRIC dataset, based on the PAM50 signature classification [33], to gain insight into the prevalence and subtype of breast cancer cases where MR and RAR signalling may be activated. While its expression is moderate, *MR* was found to be expressed widely in all breast cancer subtypes, with the highest positivity in normal-like (93%), luminal A (75%) and basal-like (74%) breast cancers (Supplementary Fig. S3D). *RARB* was less widely expressed than *MR*, its detection ranging from 16% *RARB* positive in HER2 subtype cases to 47% *RARB* positive in basal-like breast cancers. The percentage of *MR* and *RARB* double-positive detection was highest in normal-like breast cancer (40%), basal-like (35%) and luminal A breast cancer cases (14%) (Fig. 6c). As seen across the total cohort, we showed that *MR* and *RARB* double-positive cases had significantly better survival outcome in these subtypes (Fig. 6d). The basal-like subtype is characterized by poorer overall outcome (Supplementary Fig. S4B) and generally lack ER and PR, suggesting that *MR-RARB* status could add useful prognostic information for this breast cancer type.

## Discussion

This study explores the functional roles of MR in the breast and the potential crosstalk between MR and retinoic acid signalling through a combination of in silico analyses and cell line experiments. Starting with hypotheses generated from in silico analysis of NR co-expression and differential co-expression networks, which suggested (1) that MR plays a role in breast cancer biology and (2) that there is potential crosstalk between MR and RAR signalling in the normal breast, which is disrupted in breast cancer, we went on to demonstrate through expression profiling of ligand-treated MR-inducible breast cancer cells that MR and RAR signalling coordinately regulate the expression of a unique set of gene targets that are not significantly changed by either NR alone. We show here that MR and RAR signalling can inhibit both E2-dependent and E2-independent proliferation of breast cancer cells. Although it is not possible to identify which RAR mediates the effects in the cell line model since all three RAR are present, the proliferation effects are consistent with the significant association observed between high expression of both *MR* and *RARB* with better breast cancer disease-specific survival in the METABRIC breast cancer cohort. The potential relationship between *MR* and *RARB* is also supported by our original observations of a positive expression correlation between *MR* and *RARB* in the TCGA breast cancer cohort. It is possible that high expression of these two NR facilitates signalling that is ‘closer’ in biology to the normal breast, hence the more favourable outcome.

This study demonstrates the usefulness of analysing the co-expression and differential co-expression networks of NR to gain insights into their potential functional roles and generate novel hypotheses. In addition to identifying cancer-associated changes in NR regulatory networks, our investigation demonstrated that co-expression-based analysis of gene expression data is also a useful tool for the inference of potential crosstalk in the signalling networks of different NR. We showed through a combination of in silico and in vitro analyses the convergence of MR and RAR signalling, most likely through RARβ, in normal breast cells to regulate cellular metabolism and general cell maintenance which is disrupted in breast cancer due to the decreased expression of both *MR* and *RARB*. Crosstalk in MR and RARβ signalling has not been reported in the breast, although MR and RARβ are members of a group of NR reported to regulate CNS, circadian and basal metabolic response through anatomical profiling of NR expression in the mouse by Bookout et al. [34]. Our results therefore support the functional grouping of MR and RARβ observed in mouse and suggest that MR and RARβ also converge in the metabolic regulation of normal breast cells.

**Fig. 7:**
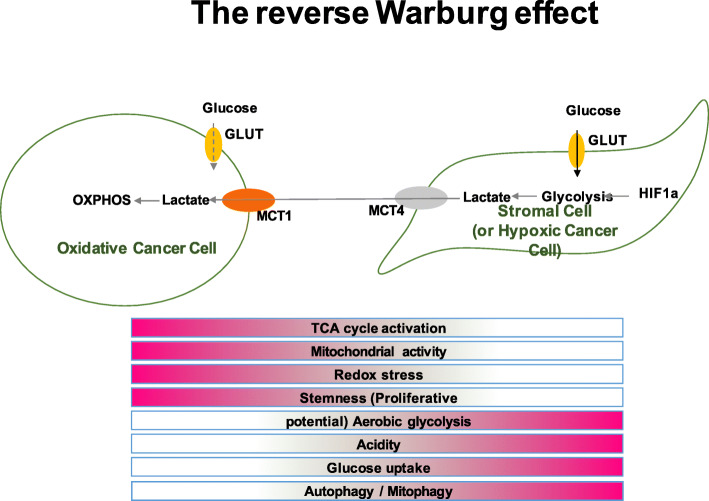
Features of the reverse Warburg effect in oxidative cancer cells and cancer-associated fibroblasts. The features of the reverse Warburg effect are shown: epithelial cancer cells induce the Warburg effect (aerobic glycolysis) in neighbouring tumour-associated fibroblasts while proliferating cancer cells themselves show a preference towards oxidative phosphorylation

While RARβ is known to suppress breast cancer through inducing apoptosis and inhibiting metastasis [25, 26, 28], relatively little is known about the role of MR in breast cancer. MR was recently reported to suppress cancer progression through suppressing the Warburg effect in a hepatocellular carcinoma cell line [35]. We showed that while co-activation of MR and RA signalling resulted in both E2-dependent and E2-independent inhibition of cell proliferation, it exerts the opposite effect on the metabolic response of breast cancer cells, specifically promoting glycolysis and suppressing oxidative phosphorylation. This is a puzzling observation being opposite to that observed in hepatocellular carcinoma. A possible explanation for this observation may lie in the ‘reverse Warburg effect’ model and the role of *HIF-1α* activation in breast cancer cells proposed by Lisanti and colleagues [36, 37]. In the reverse Warburg model, which was first observed in human breast cancer cells, epithelial cancer cells induce the Warburg effect (aerobic glycolysis) in neighbouring tumour-associated fibroblasts while proliferating cancer cells themselves show a preference towards oxidative phosphorylation. Cancer-associated fibroblasts then undergo autophagy or myo-fibroblastic differentiation, thereby secreting lactate and pyruvate (energy metabolites resulting from aerobic glycolysis) which are then taken up by the oxidative cancer cells to feed into their highly active oxidative phosphorylation processes. The characteristics of the reverse Warburg effect are summarized in Fig. 7. Indeed, co-activation of MR and RAR signalling in cells resulted in a metabolic profile resembling that reported for cancer-associated fibroblasts or hypoxic cancer cells. With this line of reasoning, the MR effect on breast cancer cell growth would still be suppressive if the reverse Warburg model is the prevalent model for breast cancer cell metabolism. Taken together, cell line modelling and human cancer sample analyses reveal that ALDO+RA treatment results in activation of HIF-1α and the hypoxia response, which promotes glycolysis and acts as a tumour suppressor in breast cancer cells, possibly due to induction of autophagy or ‘self-digestion’ of the tumour cells. Therefore, it is possible that the metabolic phenotype observed in ALDO+RA-treated cells (increased *HIF-1α* activation, increased aerobic glycolysis, decreased oxidative phosphorylation) is in fact tumour suppressive. If so, it provides mechanistic insights into the prognostic utility of MR in tamoxifen-treated breast cancers [14] and the potential for MR and RARβ to influence breast cancer cell growth through altered energy metabolism. Modulating cellular metabolic response is therefore likely one of the means through which MR and RAR signalling exert their anti-proliferative effect in breast cancer, and it will be important now to further validate our findings using additional in vitro studies.

It is likely though that in addition to the modulation of metabolic response, the tumour suppressive effect of MR is mediated through other means not fully explored in this study, including the regulation of apoptosis and angiogenesis, as suggested by the enrichment of these biological processes in genes differentially expressed under ALDO treatment in MR-inducible breast cancer cells. Finally, the suppressive effect of MR and RAR signalling in breast cancer is supported by the observation that tumours expressing high *MR* and *RARB* are associated with better survival outcome.

An intention of this study was to identify nuclear receptors that might act as novel targets or predictive factors in breast cancers, particularly those in which ER is not a viable target. While *MR* expression is on average lower in breast cancer than in normal breast tissue, we found that *MR* is present in a subset of breast cancers, and *MR-RARB* dual positivity is predictive of better breast cancer-specific survival in the METABRIC breast cancer cohort. While the MR-inducible cell line used was a breast cancer cell line (MCF-7), this choice of parent cell line partly reflects the paucity of viable non-malignant cell models in which to test our hypothesis of a functional relationship between MR and RAR signalling in the normal breast and, moreover, we would argue that given the low basal MR level of the parent, by restoring *MR* expression in these cells, the *MR*-induced state more closely resembles the normal breast or is at least closer to the good prognosis subset of breast cancer suggested by our data. Consistent with this, we observed transcriptional regulation suggestive of growth inhibition by dual ALDO and RA treatment. Importantly, the co-expression of *MR* and *RARB* was not confined to the better prognosis luminal A PAM50 subtype. Given that dual *MR-RARB* positivity was particularly prevalent in basal-like breast cancers where *ER* is seldom expressed, their expression in this breast cancer subtype holds potential clinical value.

Both ER and PR are acknowledged to contribute to breast cancer progression. Recent studies examining crosstalk between ER and PR, as well as other members of the steroid receptor family, have suggested that these NR interact at the level of DNA binding, or via converging pathway regulation, to influence breast cancer cell growth [38–41]. Both MR and the glucocorticoid receptor have been reported to crosstalk with PR in breast cancer cells to produce growth inhibitory effects [13]. Moreover, RARα has also been reported to co-operate with ER at DNA binding sites in breast cancer cells and to be positively associated with outcome in breast cancers receiving endocrine therapy [42]. Thus, interactions between MR, RARβ and ER may contribute to the positive association observed between higher MR and RARB and breast cancer disease-specific survival and the suppression of E2-mediated growth stimulation.

## Conclusions

Although less apparent, the observation that activating *MR* and *RARB* in cancer cells results in suppression of genes inversely correlated with MR and RARB in normal cells highlights the need for understanding the normal physiological functions of NR as a means of understanding their effects in breast cancer. Our study shows these receptors activate pathways independent of oestrogen action; full characterization of these pathways may identify potential targets which yield new approaches in the management of breast cancers that are refractory to treatments directly targeting the oestrogen signalling axis. This would provide new avenues for the management of breast cancers for which no effective targeted treatments exist.

## Supplementary information

**Additional file 1: Figure S1**. GO terms enriched in genes co-expressed with *MR* and *RARB* in normal breast tissues. (A) GO terms enriched in genes positively co-expressed with *MR* or *RARB* in normal breast. (B) GO terms enriched in genes negatively co-expressed with *MR* or *RARB* in normal breast tissues.

**Additional file 2: Figure S2.** GO terms enriched in genes co-expressed with *MR* and *RARB* in breast cancer tissues. (A) GO terms enriched in genes positively co-expressed with *MR* or *RARB* in breast cancer tissues. (B) GO terms enriched in genes negatively co-expressed with *MR* or *RARB* in breast cancer tissues.

**Additional file 3: Figure S3.** Expression and prevalence of *MR* and the RA receptors in the METABRIC breast cancer microarray dataset. Expression and prevalence of *MR*, *RARA*, *RARB* and *RARG* are measured in the METABRIC breast cancer microarray dataset. (A) Density plot of the distribution the expression of all probes on the array for the whole METABRIC dataset showing that the majority of probes are not expressed and therefore their expression is a measure of the baseline background probe expression. Using this plot a probe expression of value of 6 was set as a cut-off. Probes with expression > 6 was defined as being expressed. (B) Boxplot showing distribution of expression of *MR* and *RAR* transcripts in the METABRIC cohort. (C) Using the cut-off defined from (A) *MR*, *RARA*, *RARB* and *RARG* were classified as being expressed (probe expression ≥6) or not expressed (probe expression < 6) in each breast cancer sample from the METATBRIC dataset. The histogram shows the percentage of samples expressing each receptor according to this cut-off. (D) The percentage of samples within each PAM50 subtype expressing each receptor were then plotted.

**Additional file 4: Figure S4.** Survival analysis of the METABRIC breast cancer samples stratified based on *MR* and *RARB* positivity as well as PAM50 subtypes. Kaplan Meier plots of (A) All breast cancer samples from the METABRIC breast cancer microarray dataset classified based on *MR* and *RARB* positivity using probe expression cut-off defined in Fig. S3. Double-positive cases express both *MR* and *RARB* (blue); MR_only cases express *MR* but not *RARB* (yellow); RARB_only cases express *RARB* but not *MR* (red); and not_expressed cases do not express *MR* or *RARB* (grey). (B) All breast cancer samples from the METABRIC breast cancer microarray dataset stratified based on the METABRIC publication’s PAM50 classification.

**Additional file 5: Table S1.** Categories of genes, with expression correlated with MR or RARB, analysed in GO enrichment analyses.

## Data Availability

Gene expression profiling of MR-inducible MCF-7 breast cancer cells included in this article will be made available on the Gene Expression Omnibus upon publication. The METABRIC breast cancer microarrays dataset can be accessed from the European Genome-Phenome Archive (EGA) upon approval by the corresponding Data Access Committee. The TCGA RNA-Seq data used in this study is publicly available from the National Cancer Institute GDC Data Portal (https://portal.gdc.cancer.gov/).

## Abbreviations

ALDO: Aldosterone
ER: Oestrogen receptor
ER−: Oestrogen receptor negative
GO: Gene ontology
HER2: Human epidermal growth factor receptor 2
IDC: Invasive ductal carcinoma
MR: Mineralocorticoid receptor
NR: Nuclear receptor
RA: Retinoic acid
RAR: Retinoic acid receptors
SCCA: Sparse canonical correlation analysis
TLDA: Taqman low-density array
TCGA: The Cancer Genome Atlas

## References

[1] Jensen EV, Jordan VC (2003). The estrogen receptor: a model for molecular medicine. Clin Cancer Res.

[2] Mangelsdorf DJ, Thummel C, Beato M, Herrlich P, Schutz G, Umesono K, Blumberg B, Kastner P, Mark M, Chambon P, Evans RM (1995). The nuclear receptor superfamily: the second decade. Cell.

[3] Gronemeyer H, Gustafsson JA, Laudet V (2004). Principles for modulation of the nuclear receptor superfamily. Nat Rev Drug Discov.

[4] Fuller PJ (2015). Novel interactions of the mineralocorticoid receptor. Mol Cell Endocrinol.

[5] Pascual-Le Tallec L, Lombes M (2005). The mineralocorticoid receptor: a journey exploring its diversity and specificity of action. Mol Endocrinol.

[6] Fuller PJ, Yao Y, Yang J, Young MJ (2012). Mechanisms of ligand specificity of the mineralocorticoid receptor. J Endocrinol.

[7] Gomez-Sanchez EP (2016). Third-generation mineralocorticoid receptor antagonists: why do we need a fourth?. J Cardiovasc Pharmacol.

[8] Ahmed AH, Gordon RD, Taylor PJ, Ward G, Pimenta E, Stowasser M (2011). Are women more at risk of false-positive primary aldosteronism screening and unnecessary suppression testing than men?. J Clin Endocrinol Metab.

[9] Gomez-Sanchez E, Gomez-Sanchez CE (2014). The multifaceted mineralocorticoid receptor. Compr Physiol.

[10] Pippal JB, Yao Y, Rogerson FM, Fuller PJ (2009). Structural and functional characterization of the interdomain interaction in the mineralocorticoid receptor. Mol Endocrinol.

[11] Fuller PJ, Yang J, Young MJ (2017). 30 years of the mineralocorticoid receptor: coregulators as mediators of mineralocorticoid receptor signalling diversity. J Endocrinol.

[12] Voutsadakis IA (2016). Epithelial-mesenchymal transition (EMT) and regulation of EMT factors by steroid nuclear receptors in breast cancer: a review and in silico investigation. J Clin Med..

[13] Leo JCL, Guo C, Woon CT, Aw SE, Lin VCL (2004). Glucocorticoid and mineralocorticoid cross-talk with progesterone receptor to induce focal adhesion and growth inhibition in breast cancer cells. Endocrinology.

[14] Muscat GE, Eriksson NA, Byth K, Loi S, Graham D, Jindal S, Davis MJ, Clyne C, Funder JW, Simpson ER, Ragan MA, Kuczek E, Fuller PJ, Tilley WD, Leedman PJ, Clarke CL (2013). Research resource: nuclear receptors as transcriptome: discriminant and prognostic value in breast cancer. Mol Endocrinol.

[15] Doan TB, Eriksson NA, Graham D, Funder JW, Simpson ER, Kuczek ES, Clyne C, Leedman PJ, Tilley WD, Fuller PJ, Muscat GE, Clarke CL (2014). Breast cancer prognosis predicted by nuclear receptor-coregulator networks. Mol Oncol.

[16] Vandesompele J, De Preter K, Pattyn F, Poppe B, Van Roy N, De Paepe A, Speleman F. Accurate normalization of real-time quantitative RT-PCR data by geometric averaging of multiple internal control genes. Genome Biol Res. 2002;3(7): 0034.1. 10.1186/gb-2002-3-7-research0034 PMC126239 12184808

[17] Du P, Kibbe WA, Lin SM (2008). lumi: a pipeline for processing Illumina microarray. Bioinformatics..

[18] Ritchie ME, Phipson B, Wu D, Hu Y, Law CW, Shi W, Smyth GK (2015). limma powers differential expression analyses for RNA-sequencing and microarray studies. Nucleic Acids Res.

[19] Huang DW, Sherman BT, Lempicki RA (2009). Systematic and integrative analysis of large gene lists using DAVID bioinformatics resources. Nat Protoc.

[20] Huang DW, Sherman BT, Lempicki RA (2009). Bioinformatics enrichment tools: paths toward the comprehensive functional analysis of large gene lists. Nucleic Acids Res.

[21] Curtis C, Shah SP, Chin SF, Turashvili G, Rueda OM, Dunning MJ, Speed D, Lynch AG, Samarajiwa S, Yuan Y, Graf S, Ha G, Haffari G, Bashashati A, Russell R, McKinney S, Langerod A, Green A, Provenzano E, Wishart G, Pinder S, Watson P, Markowetz F, Murphy L, Ellis I, Purushotham A, Borresen-Dale AL, Brenton JD, Tavare S, Caldas C, Aparicio S (2012). The genomic and transcriptomic architecture of 2,000 breast tumours reveals novel subgroups. Nature.

[22] Parkhomenko E, Tritchler D, Beyene J: Sparse canonical correlation analysis with application to genomic data integration. Stat Applications in Genet Molecular Biol 2009, 8:Article 1. 10.2202/1544-6115.1406 19222376

[23] Holbeck S, Chang J, Best AM, Bookout AL, Mangelsdorf DJ, Martinez ED (2010). Expression profiling of nuclear receptors in the NCI60 cancer cell panel reveals receptor-drug and receptor-gene interactions. Mol Endocrinol.

[24] Warburg O (1956). On the origin of cancer cells. Science.

[25] Flamini MI, Gauna GV, Sottile ML, Nadin BS, Sanchez AM, Vargas-Roig LM (2014). Retinoic acid reduces migration of human breast cancer cells: role of retinoic acid receptor beta. J Cell Mol Med.

[26] Liu Y, Lee MO, Wang HG, Li Y, Hashimoto Y, Klaus M, Reed JC, Zhang X (1996). Retinoic acid receptor beta mediates the growth-inhibitory effect of retinoic acid by promoting apoptosis in human breast cancer cells. Mol Cell Biol.

[27] Sun SY, Wan H, Yue P, Hong WK, Lotan R (2000). Evidence that retinoic acid receptor beta induction by retinoids is important for tumor cell growth inhibition. J Biol Chem.

[28] Treuting PM, Chen LI, Buetow BS, Zeng W, Birkebak TA, Seewaldt VL, Sommer KM, Emond M, Maggio-Price L, Swisshelm K (2002). Retinoic acid receptor beta2 inhibition of metastasis in mouse mammary gland xenografts. Breast Cancer Res Treat.

[29] Xu XC, Sneige N, Liu X, Nandagiri R, Lee JJ, Lukmanji F, Hortobagyi G, Lippman SM, Dhingra K, Lotan R (1997). Progressive decrease in nuclear retinoic acid receptor beta messenger RNA level during breast carcinogenesis. Cancer Res.

[30] Ward PS, Thompson CB (2012). Metabolic reprogramming: a cancer hallmark even Warburg did not anticipate. Cancer Cell.

[31] Munoz-Pinedo C, El Mjiyad N, Ricci JE (2012). Cancer metabolism: current perspectives and future directions. Cell Death and Disease.

[32] Venet D, Dumont JE, Detours V: Most random gene expression signatures are significantly associated with breast cancer outcome. PLoS Comput Biol 2011, 7(10). 10.1371/journal.pcbi.1002240 PMC3197658 22028643

[33] Parker JS, Mullins M, Cheang MC, Leung S, Voduc D, Vickery T, Davies S, Fauron C, He X, Hu Z, Quackenbush JF, Stijleman IJ, Palazzo J, Marron JS, Nobel AB, Mardis E, Ellis MJ, Perou CM, Bernard PS, Nielsen TO (2009). Supervised risk predictor of breast cancer based on intrinsic subtypes. J Clin Oncol.

[34] Bookout AL, Jeong Y, Downes M, Yu RT, Evans RM, Mangelsdorf DJ: Anatomical profiling of nuclear receptor expression reveals a hierarchical transcriptional network. 2006, 126(4):789–799. 10.1016/j.cell.2006.06.049 PMC6211849 16923397

[35] Nie H, Li J, Yang XM, Cao QZ, Feng MX, Xue F, Wei L, Qin W, Gu J, Xia Q, Zhang ZG (2015). Mineralocorticoid receptor suppresses cancer progression and the Warburg effect by modulating the miR-338-3p-PKLR axis in hepatocellular carcinoma. Hepatology.

[36] Chiavarina B, Whitaker-Menezes D, Migneco G, Martinez-Outschoorn UE, Pavlides S, Howell A, Tanowitz HB, Casimiro MC, Wang C, Pestell RG, Grieshaber P, Caro J, Sotgia F, Lisanti MP (2010). HIF1-alpha functions as a tumor promoter in cancer associated fibroblasts, and as a tumor suppressor in breast cancer cells: autophagy drives compartment-specific oncogenesis. Cell Cycle.

[37] Pavlides S, Whitaker-Menezes D, Castello-Cros R, Flomenberg N, Witkiewicz AK, Frank PG, Casimiro MC, Wang C, Fortina P, Addya S, Pestell RG, Martinez-Outschoorn UE, Sotgia F, Lisanti MP (2009). The reverse Warburg effect: aerobic glycolysis in cancer associated fibroblasts and the tumor stroma. Cell Cycle.

[38] Daniel AR, Gaviglio AL, Knutson TP, Ostrander JH, D'Assoro AB, Ravindranathan P, Peng Y, Raj GV, Yee D, Lange CA (2015). Progesterone receptor-B enhances estrogen responsiveness of breast cancer cells via scaffolding PELP1- and estrogen receptor-containing transcription complexes. Oncogene.

[39] Hilton HN, Doan TB, Graham JD, Oakes SR, Silvestri A, Santucci N, Kantimm S, Huschtscha LI, Ormandy CJ, Funder JW, Simpson ER, Kuczek ES, Leedman PJ, Tilley WD, Fuller PJ, Muscat GE, Clarke CL (2014). Acquired convergence of hormone signaling in breast cancer: ER and PR transition from functionally distinct in normal breast to predictors of metastatic disease. Oncotarget.

[40] Mohammed H, Russell IA, Stark R, Rueda OM, Hickey TE, Tarulli GA, Serandour AA, Birrell SN, Bruna A, Saadi A, Menon S, Hadfield J, Pugh M, Raj GV, Brown GD, D'Santos C, Robinson JL, Silva G, Launchbury R, Perou CM, Stingl J, Caldas C, Tilley WD, Carroll JS (2015). Progesterone receptor modulates ERalpha action in breast cancer. Nature.

[41] Sikora MJ (2016). Family matters: collaboration and conflict among the steroid receptors raises a need for group therapy. Endocrinology.

[42] Ross-Innes CS, Stark R, Holmes KA, Schmidt D, Spyrou C, Russell R, Massie CE, Vowler SL, Eldridge M, Carroll JS (2010). Cooperative interaction between retinoic acid receptor-α and estrogen receptor in breast cancer. Genes Dev.

