# Supplementary material for: A tumour suppressive relationship between mineralocorticoid and retinoic acid receptors activates a transcriptional program consistent with a reverse Warburg effect in breast cancer
Source: Breast Cancer Res. 2020 Nov 4;22:122. doi: 10.1186/s13058-020-01355-x (PMC7641839; doi:10.1186/s13058-020-01355-x)
Supplement: Supplementary file 1 — Additional file 1: Figure S1. GO terms enriched in genes co-expressed with MR and RARB in normal breast tissues. (A) GO terms enriched in genes positively co-expressed with MR or RARB in normal breast. (B) GO terms enriched in genes negatively co-expressed with MR or RARB in normal breast tissues. [file 13058_2020_1355_MOESM1_ESM.pdf]

Figure S1

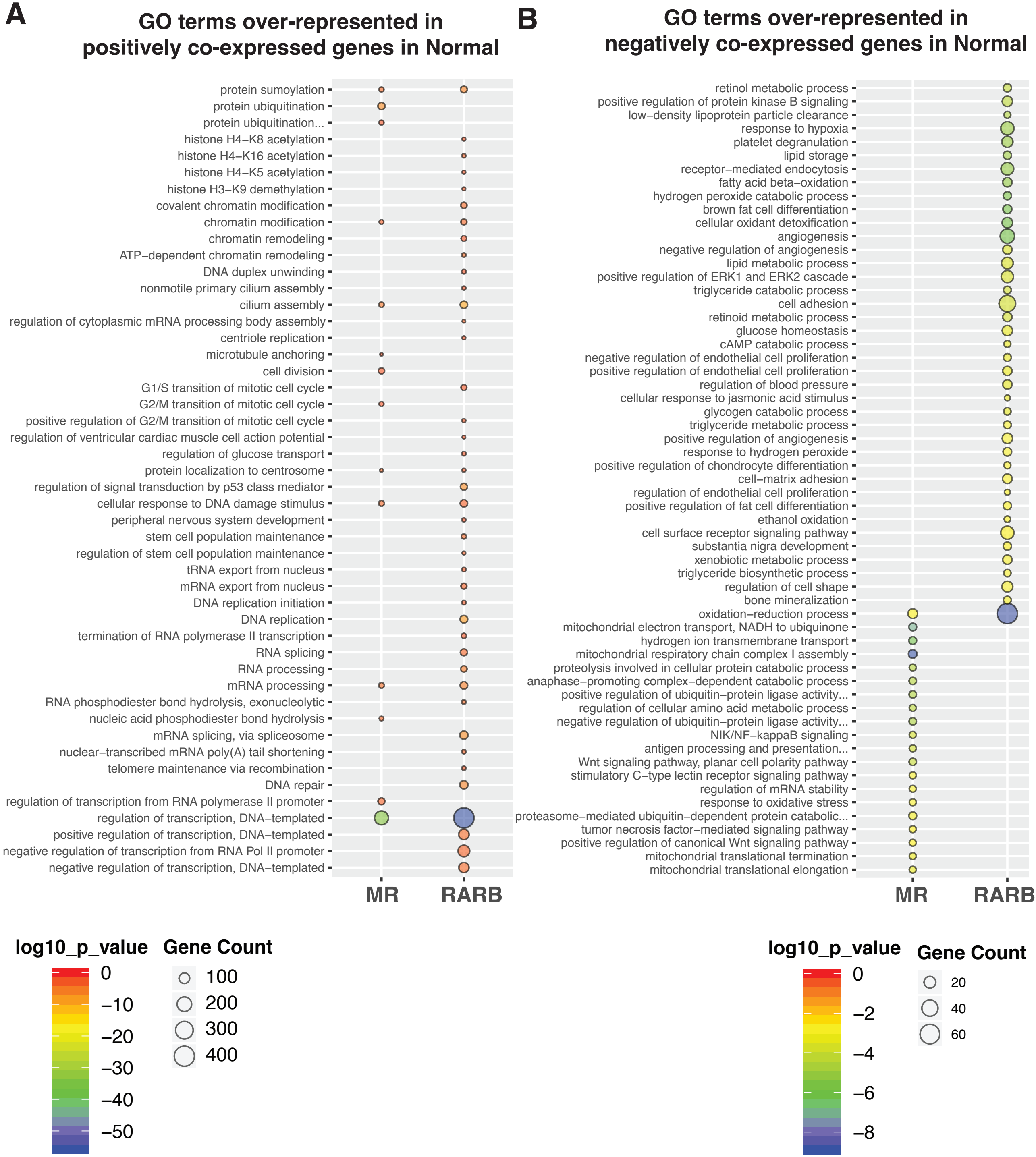
A
GO terms over-represented in positively co-expressed genes in Normal
protein sumoylation
protein ubiquitination
protein ubiquitination...
histone H4−K8 acetylation
histone H4−K16 acetylation
histone H4−K5 acetylation
histone H3−K9 demethylation
covalent chromatin modification
chromatin modification
chromatin remodeling
ATP−dependent chromatin remodeling
DNA duplex unwinding
nonmotile primary cilium assembly
cilium assembly
regulation of cytoplasmic mRNA processing body assembly
centriole replication
microtubule anchoring
cell division
G1/S transition of mitotic cell cycle
G2/M transition of mitotic cell cycle
positive regulation of G2/M transition of mitotic cell cycle
regulation of ventricular cardiac muscle cell action potential
regulation of glucose transport
protein localization to centrosome
regulation of signal transduction by p53 class mediator
cellular response to DNA damage stimulus
peripheral nervous system development
stem cell population maintenance
regulation of stem cell population maintenance
tRNA export from nucleus
mRNA export from nucleus
DNA replication initiation
DNA replication
termination of RNA polymerase II transcription
RNA splicing
RNA processing
mRNA processing
RNA phosphodiester bond hydrolysis, exonucleolytic
nucleic acid phosphodiester bond hydrolysis
mRNA splicing, via spliceosome
nuclear−transcribed mRNA poly(A) tail shortening
telomere maintenance via recombination
DNA repair
regulation of transcription from RNA polymerase II promoter
regulation of transcription, DNA−templated
positive regulation of transcription, DNA−templated
negative regulation of transcription from RNA Pol II promoter
negative regulation of transcription, DNA−templated
MR
RARB
log10_p_value
0
−10
−20
−30
−40
−50
Gene Count
100
200
300
400
B
GO terms over-represented in negatively co-expressed genes in Normal
retinol metabolic process
positive regulation of protein kinase B signaling
low−density lipoprotein particle clearance
response to hypoxia
platelet degranulation
lipid storage
receptor−mediated endocytosis
fatty acid beta−oxidation
hydrogen peroxide catabolic process
brown fat cell differentiation
cellular oxidant detoxification
angiogenesis
negative regulation of angiogenesis
lipid metabolic process
positive regulation of ERK1 and ERK2 cascade
triglyceride catabolic process
cell adhesion
retinoid metabolic process
glucose homeostasis
cAMP catabolic process
negative regulation of endothelial cell proliferation
positive regulation of endothelial cell proliferation
regulation of blood pressure
cellular response to jasmonic acid stimulus
glycogen catabolic process
triglyceride metabolic process
positive regulation of angiogenesis
response to hydrogen peroxide
positive regulation of chondrocyte differentiation
cell−matrix adhesion
regulation of endothelial cell proliferation
positive regulation of fat cell differentiation
ethanol oxidation
cell surface receptor signaling pathway
substantia nigra development
xenobiotic metabolic process
triglyceride biosynthetic process
regulation of cell shape
bone mineralization
oxidation−reduction process
mitochondrial electron transport, NADH to ubiquinone
hydrogen ion transmembrane transport
mitochondrial respiratory chain complex I assembly
proteolysis involved in cellular protein catabolic process
anaphase−promoting complex−dependent catabolic process
positive regulation of ubiquitin−protein ligase activity...
regulation of cellular amino acid metabolic process
negative regulation of ubiquitin−protein ligase activity...
NIK/NF−kappaB signaling
antigen processing and presentation...
Wnt signaling pathway, planar cell polarity pathway
stimulatory C−type lectin receptor signaling pathway
regulation of mRNA stability
response to oxidative stress
proteasome−mediated ubiquitin−dependent protein catabolic...
tumor necrosis factor−mediated signaling pathway
positive regulation of canonical Wnt signaling pathway
mitochondrial translational termination
mitochondrial translational elongation
MR
RARB
log10_p_value
0
−2
−4
−6
−8
Gene Count
20
40
60
