# Supplementary material for: A tumour suppressive relationship between mineralocorticoid and retinoic acid receptors activates a transcriptional program consistent with a reverse Warburg effect in breast cancer
Source: Breast Cancer Res. 2020 Nov 4;22:122. doi: 10.1186/s13058-020-01355-x (PMC7641839; doi:10.1186/s13058-020-01355-x)
Supplement: Supplementary file 2 — Additional file 2: Figure S2. GO terms enriched in genes co-expressed with MR and RARB in breast cancer tissues. (A) GO terms enriched in genes positively co-expressed with MR or RARB in breast cancer tissues. (B) GO terms enriched in genes negatively co-expressed with MR or RARB in breast cancer tissues. [file 13058_2020_1355_MOESM2_ESM.pdf]

Figure S2

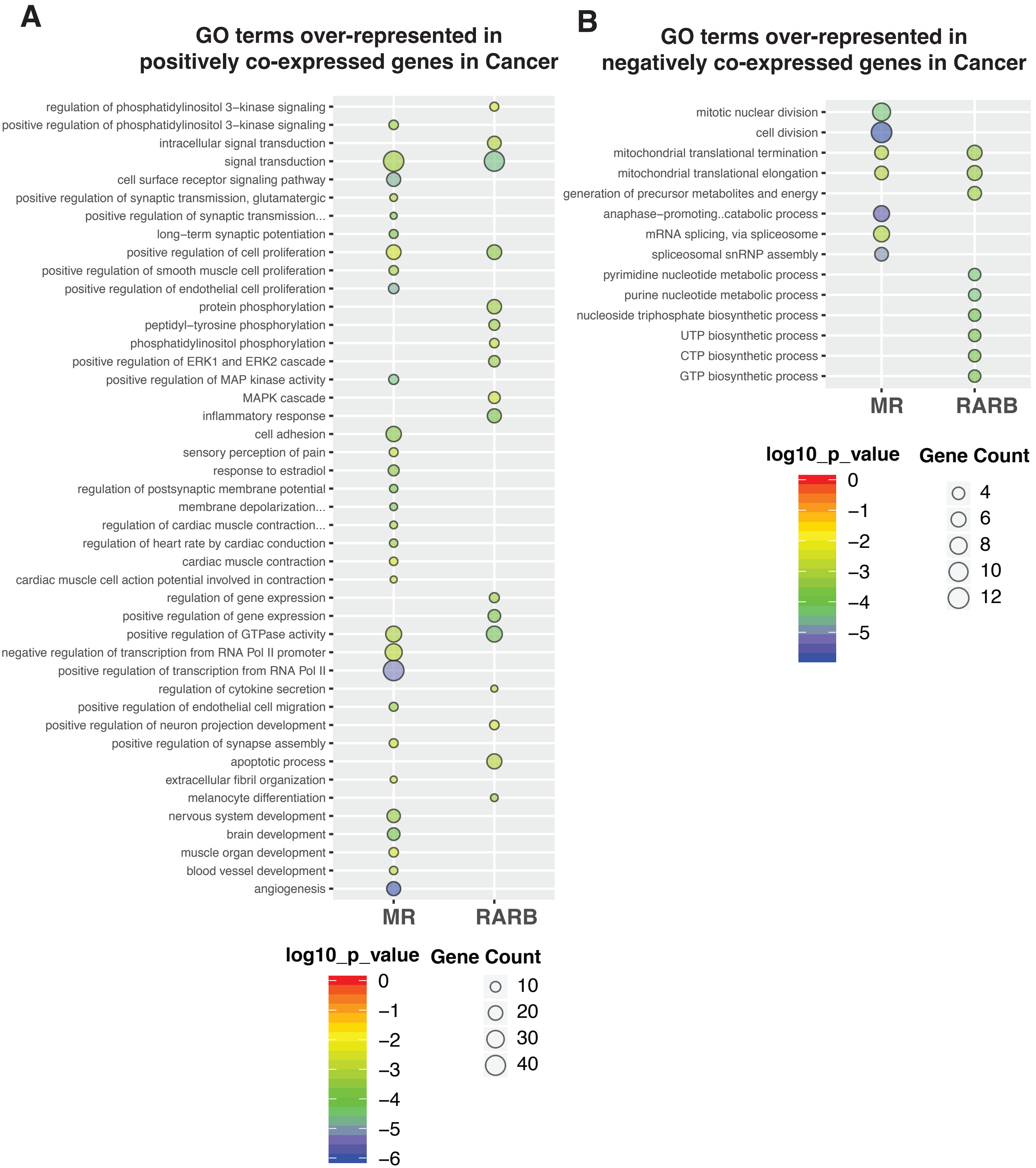

A
GO terms over-represented in positively co-expressed genes in Cancer
regulation of phosphatidylinositol 3−kinase signaling
positive regulation of phosphatidylinositol 3−kinase signaling
intracellular signal transduction
signal transduction
cell surface receptor signaling pathway
positive regulation of synaptic transmission, glutamatergic
positive regulation of synaptic transmission...
long−term synaptic potentiation
positive regulation of cell proliferation
positive regulation of smooth muscle cell proliferation
positive regulation of endothelial cell proliferation
protein phosphorylation
peptidyl−tyrosine phosphorylation
phosphatidylinositol phosphorylation
positive regulation of ERK1 and ERK2 cascade
positive regulation of MAP kinase activity
MAPK cascade
inflammatory response
cell adhesion
sensory perception of pain
response to estradiol
regulation of postsynaptic membrane potential
membrane depolarization...
regulation of cardiac muscle contraction...
regulation of heart rate by cardiac conduction
cardiac muscle contraction
cardiac muscle cell action potential involved in contraction
regulation of gene expression
positive regulation of gene expression
positive regulation of GTPase activity
negative regulation of transcription from RNA Pol II promoter
positive regulation of transcription from RNA Pol II
regulation of cytokine secretion
positive regulation of endothelial cell migration
positive regulation of neuron projection development
positive regulation of synapse assembly
apoptotic process
extracellular fibril organization
melanocyte differentiation
nervous system development
brain development
muscle organ development
blood vessel development
angiogenesis
MR
RARB
log10_p_value
0
−1
−2
−3
−4
−5
−6
Gene Count
10
20
30
40
B
GO terms over-represented in negatively co-expressed genes in Cancer
mitotic nuclear division
cell division
mitochondrial translational termination
mitochondrial translational elongation
generation of precursor metabolites and energy
anaphase−promoting..catabolic process
mRNA splicing, via spliceosome
spliceosomal snRNP assembly
pyrimidine nucleotide metabolic process
purine nucleotide metabolic process
nucleoside triphosphate biosynthetic process
UTP biosynthetic process
CTP biosynthetic process
GTP biosynthetic process
MR
RARB
log10_p_value
0
−1
−2
−3
−4
−5
Gene Count
4
6
8
10
12
