# Supplementary figures and images for: A tumour suppressive relationship between mineralocorticoid and retinoic acid receptors activates a transcriptional program consistent with a reverse Warburg effect in breast cancer
Source: Breast Cancer Res. 2020 Nov 4;22:122. doi: 10.1186/s13058-020-01355-x (PMC7641839; doi:10.1186/s13058-020-01355-x)

**A**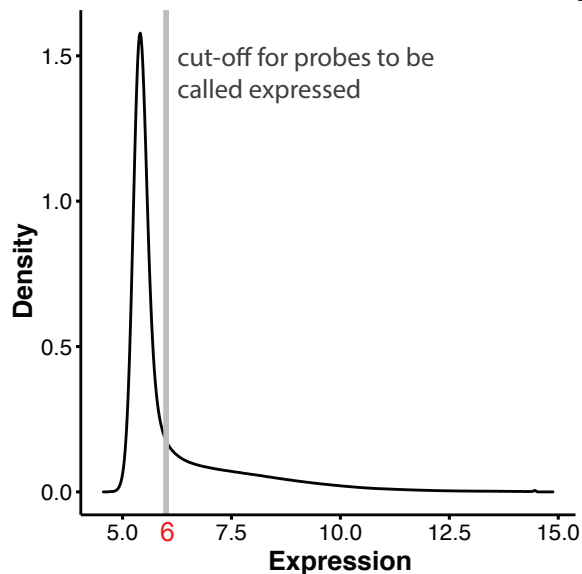**B**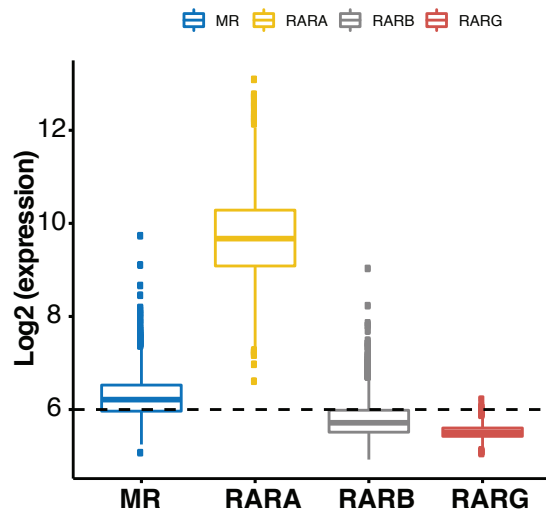**C**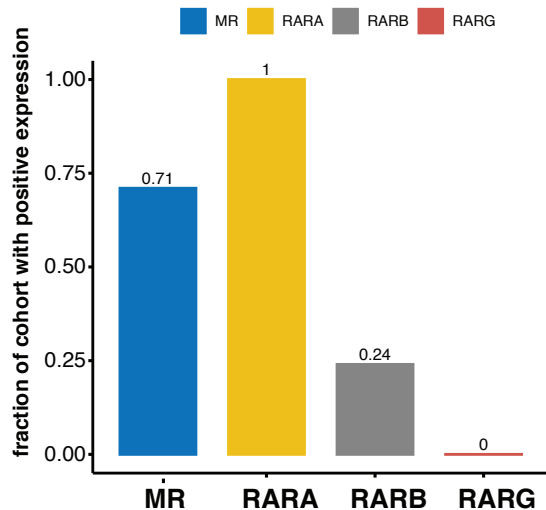**D**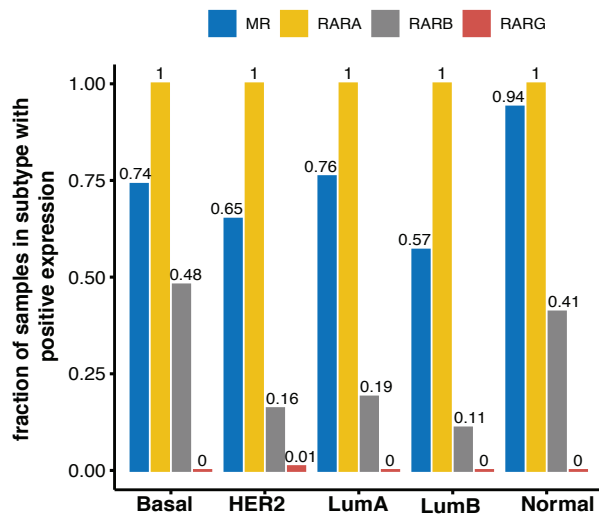

Supplement: Supplementary file 3 — Additional file 3: Figure S3. Expression and prevalence of MR and the RA receptors in the METABRIC breast cancer microarray dataset. Expression and prevalence of MR, RARA, RARB and RARG are measured in the METABRIC breast cancer microarray dataset. (A) Density plot of the distribution the expression of all probes on the array for the whole METABRIC dataset showing that the majority of probes are not expressed and therefore their expression is a measure of the baseline background probe expression. Using this plot a probe expression of value of 6 was set as a cut-off. Probes with expression > 6 was defined as being expressed. (B) Boxplot showing distribution of expression of MR and RAR transcripts in the METABRIC cohort. (C) Using the cut-off defined from (A) MR, RARA, RARB and RARG were classified as being expressed (probe expression ≥6) or not expressed (probe expression < 6) in each breast cancer sample from the METATBRIC dataset. The histogram shows the percentage of samples expressing each receptor according to this cut-off. (D) The percentage of samples within each PAM50 subtype expressing each receptor were then plotted. [file 13058_2020_1355_MOESM3_ESM.pdf]

**A**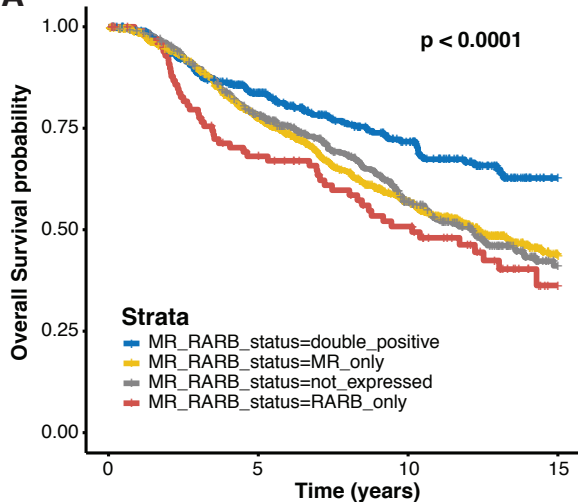**B**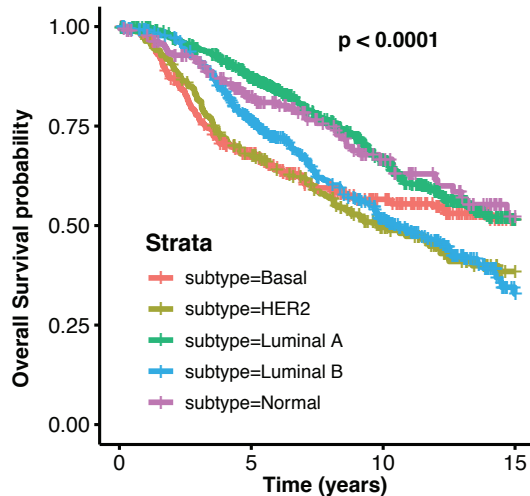

Supplement: Supplementary file 4 — Additional file 4: Figure S4. Survival analysis of the METABRIC breast cancer samples stratified based on MR and RARB positivity as well as PAM50 subtypes. Kaplan Meier plots of (A) All breast cancer samples from the METABRIC breast cancer microarray dataset classified based on MR and RARB positivity using probe expression cut-off defined in Fig. S3. Double-positive cases express both MR and RARB (blue); MR_only cases express MR but not RARB (yellow); RARB_only cases express RARB but not MR (red); and not_expressed cases do not express MR or RARB (grey). (B) All breast cancer samples from the METABRIC breast cancer microarray dataset stratified based on the METABRIC publication’s PAM50 classification. [file 13058_2020_1355_MOESM4_ESM.pdf]
