# Supplementary material for: A tumour suppressive relationship between mineralocorticoid and retinoic acid receptors activates a transcriptional program consistent with a reverse Warburg effect in breast cancer
Source: Breast Cancer Res. 2020 Nov 4;22:122. doi: 10.1186/s13058-020-01355-x (PMC7641839; doi:10.1186/s13058-020-01355-x)
Supplement: Supplementary file 5 — Additional file 5: Table S1. Categories of genes, with expression correlated with MR or RARB, analysed in GO enrichment analyses. [file 13058_2020_1355_MOESM5_ESM.docx]

**Supplementary Table 1:** Categories of genes, with expression correlated with MR or RARB, analysed in GO enrichment analyses.

| **Category** | **Numbers** |
| --- | --- |
| Genes positively correlated with MR in normal breast | 917 |
| Genes positively correlated with MR in breast cancer | 395 |
| Genes positively correlated with RARB in normal breast | 2518 |
| Genes positively correlated with RARB in breast cancer | 330 |
| Genes negatively correlated with MR in normal breast | 144 |
| Genes negatively correlated with MR in breast cancer | 102 |
| Genes negatively correlated with RARB in normal breast | 928 |
| Genes negatively correlated with RARB in breast cancer | 146 |
